# GPR4 signaling is essential for the promotion of acid-mediated angiogenic capacity of endothelial progenitor cells by activating STAT3/VEGFA pathway in patients with coronary artery disease

**DOI:** 10.1186/s13287-021-02221-z

**Published:** 2021-02-25

**Authors:** Shun Ouyang, Yan Li, Xing Wu, Yan Wang, Fanmao Liu, Jianning Zhang, Yumin Qiu, Zhe Zhou, Zhichao Wang, Wenhao Xia, Xiufang Lin

**Affiliations:** 1 Department of Cardiology, The Fifth Affiliated Hospital, Sun Yat-Sen University, Zhuhai, 519000 China; 2 Department of Cardiovascular Medicine, The First Affiliated Hospital of Jinan University, Guangzhou, 510630 China; 3 Department of Hypertension and Vascular Disease, The First Affiliated Hospital, Sun Yat-Sen University, Guangzhou, 510080 China

**Keywords:** Coronary artery disease, Acidic microenvironment, Angiogenesis, GPR4, STAT3, EPCs

## Abstract

**Background:**

Patients with coronary artery disease (CAD) are characterized by a decline in vascular regeneration, which is related to the dysfunction of endothelial progenitor cells (EPCs). G-protein-coupled receptor 4 (GPR4) is a proton-sensing G-protein-coupled receptor (GPCR) that contributes to neovascularization in acidic microenvironments. However, the role of GPR4 in regulating the angiogenic capacity of EPCs from CAD patients in response to acidity generated in ischemic tissue remains completely unclear.

**Methods:**

The angiogenic capacity of EPCs collected from CAD patients and healthy subjects was evaluated in different pH environments. The GPR4 function of regulating EPC-mediated angiogenesis was analyzed both in vitro and in vivo. The downstream mechanisms were further investigated by genetic overexpression and inhibition.

**Results:**

Acidic environment prestimulation significantly enhanced the angiogenic capacity of EPCs from the non-CAD group both in vivo and in vitro, while the same treatment yielded the opposite result in the CAD group. Among the four canonical proton-sensing GPCRs, GPR4 displays the highest expression in EPCs. The expression of GRP4 was markedly lower in EPCs from CAD patients than in EPCs from non-CAD individuals independent of acid stimulation. The siRNA-mediated knockdown of GPR4 with subsequent decreased phosphorylation of STAT3 mimicked the impaired function of EPCs from CAD patients at pH 6.4 but not at pH 7.4. Elevating GPR4 expression restored the neovessel formation mediated by EPCs from CAD patients in an acidic environment by activating STAT3/VEGFA signaling. Moreover, the beneficial impact of GPR4 upregulation on EPC-mediated angiogenic capacity was abrogated by blockade of the STAT3/VEGFA signaling pathway.

**Conclusions:**

Our present study demonstrated for the first time that loss of GPR4 is responsible for the decline in proton sensing and angiogenic capacity of EPCs from CAD patients. Augmentation of GPR4 expression promotes the neovessel formation of EPCs by activating STAT3/VEGF signaling. This finding implicates GPR4 as a potential therapeutic target for CAD characterized by impaired neovascularization in ischemic tissues.

**Supplementary Information:**

The online version contains supplementary material available at 10.1186/s13287-021-02221-z.

## Background

Coronary artery disease (CAD) is a major cause of death globally. The disruption in blood supply induced by a partial or complete blockage of coronary arteries causes myocardial ischemia, myocardial infarction (MI), and subsequent deterioration in function [1, 2]. Restoring blood supply to sites of myocardial ischemia by efficient development of new vessels could reduce myocardial necrosis and substantially improve the outcome of CAD patients. Previous research has shown that endothelial progenitor cells (EPCs) contribute to the repair of myocardial damage by recruiting to ischemic sites and promoting vascular rejuvenation [3]. However, the mechanisms controlling EPC-mediated neovascularization in the ischemic microenvironment remain largely unknown.

Due to poor perfusion, anaerobic glycolysis dominates the metabolic process and gives rise to an increase in lactate and H+ concentrations, leading to local acidosis at ischemic sites [4, 5]. The mammalian coronary ligation model represents a decrease in the pH from 7.4 to ∼5.5 [6]. Hence, EPCs from CAD patients homing to ischemic sites are exposed to an acidic microenvironment. The acidic microenvironment may cause extracellular matrix degradation, attenuate immune responses, and modify cellular and intercellular signaling [7, 8]. Acidic extracellular pH has pleiotropic effects on the angiogenesis process [9]. Recent work has demonstrated that acidic preconditioning greatly improves endothelial colony-forming cell survival and angiogenic activity at ischemic sites [9, 10]. However, the poor responsivity to acidity of the migrated and local EPCs within the injured tissue is an additional limitation for revascularization in sites myocardial ischemia in CAD patients.

G-protein-coupled receptor 4 (GPR4) is a pH-sensing G-protein-coupled receptor that is highly expressed in vascular endothelial cells and can be activated by protons in the ischemic microenvironment [11]. Previous studies have demonstrated that GPR4 can promote angiogenesis at acidic pH [12] and is capable of regulating the tube formation, migration, and proliferation of ECs [13]. GPR4 knockout mice displayed altered vessel morphology and reduced vascular length and density. Moreover, GPR4 deficiency results in reduced VEGF receptor 2 (VEGF2) levels in endothelial cells. These results suggested that GPR4 is required for endothelial cell-mediated vascular regeneration [14]. However, little is known about whether GPR4 mediates the responsivity to acidity generated in the ischemic tissue and angiogenic capacity of EPCs from patients with CAD.

In this study, we hypothesized that GPR4 is a vital marker that modulates the extracellular pH response of EPCs and that elevated GPR4 expression can improve the angiogenic function of EPCs from CAD patients. To address these hypotheses, the expression level of GPR4 on EPCs from healthy controls and CAD patients was evaluated. Then, both in vitro function and in vivo angiogenic capacity in a nude mouse model of hindlimb ischemia after enhancing the expression of GPR4 in EPCs were examined. From the new perspective of chemicobiology, the present study may identify GPR4 as a key sensor of the acidic microenvironment of EPCs in ischemic tissues, which could be targeted for the prevention and treatment of CAD.

## Materials and methods

### Isolation of peripheral blood mononuclear cells (PBMNCs), late EPC culture, and flow cytometry analysis

PBMNCs were isolated by Ficoll density gradient centrifugation from healthy subjects and CAD patients as described previously, and late EPCs were cultured and characterized by following the protocol described [15, 16]. In short, PBMCs were cultured on fibronectin-coated 6-well plates in EBM-2 (contents: ascorbic acid 0.5 ml, rhFGF-B 2.0 ml, heparin 0.5 ml, GA-1000 0.5 ml, rhEGF 0.5 ml, hydrocortisone 0.2 ml, VEGF 0.5 ml, R3-IGF-1 0.5 ml) supplemented with endothelial growth medium-SingleQuots (Clonetics, San Diego, CA, USA). For all assays, late EPCs were used at passage 3 (approximately 28 days) after being removed by complete washing with culture medium. After 28 days, endothelial markers of cultured EPCs were examined by flow cytometry analysis using CD31 (BD Biosciences, San Jose, CA, USA), Tie-2 receptor (BD Biosciences), and kinase-insert domain receptor (KDR) (R&D Systems, Inc., Minneapolis, MN, USA). EPCs isolated from healthy and CAD patients separately were both cultured at 37 °C and 5% CO_2_ in EBM-2, and the medium was changed every 48 h.

### Late EPC angiogenesis in vitro

The angiogenic capacity of EPCs was determined using the Matrigel tube-like formation test [17]. Matrigel and EGM-2 media were polymerized in 96-well plates at 37 °C and 5% CO_2_ for 1 h. P2 generation cells were resuspended in EBM-2 and loaded on top of the Matrigel. Each conditional group contained 3 wells. Following incubation at 37 °C for 4 h. Finally, the cells were observed, and images were taken by optical microscopy (Canon, Japan). The network area and length were evaluated with ImageJ software. Similarly, the acid-treated EPCs were treated with gene transfer technology and siRNA and then subjected to Matrigel experiments (Table 1).

**Table 1:** Clinical characteristics of the recruited subjects

Parameters	Control group (*n* = 10)	CAD group (*n* = 10)	*P* value
Age (years)	51.3 ± 6.1	50.6 ± 7.8	0.45
Male	4 (40%)	5 (50%)	0.65
BMI (kg/m^2^)	23.8 ± 0.8	24.9 ± 1.3	0.18
Systolic blood pressure (mmHg)	125.8 ± 7.6	117.0 ± 11.1	0.37
Diastolic blood pressure (mmHg)	72.3 ± 8.6	72.6 ± 8.4	0.86
Fasting plasma glucose (mmol/L)	4.7 ± 0.6	5.6 ± 0.8	0.10
Diabetes	1 (10%)	2 (20%)	0.53
Current smoking	2 (20%)	4 (40%)	0.33
Total cholesterol (mmol/L)	3.7 ± 0.5	4.9 ± 0.9	0.22
Triglyceride (mmol/L)	1.1 ± 0.4	1.3 ± 0.5	0.93
HDL cholesterol (mmol/L)	1.1 ± 0.2	1.2 ± 0.3	0.16
LDL cholesterol (mmol/L)	2.2 ± 0.3	3.0 ± 0.6	0.09
Creatinine (μmol/L)	76.6 ± 13.2	71.9 ± 13.5	0.80

*BMI* body mass index, *CAD* coronary artery disease, *HDL* high-density lipoprotein, *LDL* low-density lipoprotein

### Late EPC migration in vitro

EPC migration was determined by the wound healing assay. A total of 2 × 10^5^ P2-generation EPCs were plated in 6-well culture dishes. The wound was created by manually scraping the cell monolayer with a p20 pipette tip, washing once with PBS solution and supplementing 2 ml EBM-2. After 24 h of incubation at 37 °C, transmigrated cells were counted. Similarly, the acid-treated EPCs were treated with gene transfer technology and siRNA, and then, wound healing experiments were performed.

### Acid stimulation

To prepare isocapnic pH media, EGM-2 was first buffered with a 7.5-mM HEPES, and the pH of the media was then measured with an electronic pH meter (Unisense, Aarhus, Denmark) using NaOH or HCl to adjust to the desired pH. To prepare appropriate media, regular EGM-2 medium was incubated for at least 1 h in humidified tissue culture incubators with 5% CO_2_ or 20% CO_2_. After that, the pH of each medium was measured to be approximately 7.4 and 6.4.

### EPC gene transfer

After 28 days in culture, cells were transduced with pcDNA3.1(+) encoding the human GPR4 gene (pcDNA3.1(+)-GPR4), a vector (pcDNA3.1(+) H302), or the enhanced green fluorescent protein gene (H312 pEGFP-N1) (Obio Technology, Shanghai, China). H312 pEGFP-N1 was used only to detect transfection efficiency by fluorescence microscopy. Neither pcDNA3.1(+)-GPR4 nor the vector contained the enhanced green fluorescent protein gene as determined by western blot and subsequent experiments. Human EPCs were transduced with the pcDNA3.1(+)-GPR4, vector (pcDNA3.1(+) H302), or H312 pEGFP-N1 for 90 min in culture medium without serum. After transduction, the cells were washed with PBS and incubated with acidic medium for 6 h before being used in subsequent experiments.

### siRNA transfection

EPCs were transfected with a 5-μl GPR4-siRNA or 5-μl scramble siRNA (RiboBio Technology, Shanghai, China) for 48 h by using Lipofectamine® RNAiMAX reagent (Thermo Fisher Scientific, MA, USA) according to the manufacturer’s protocol before treatment with acidic medium. After transduction, the cells were washed with PBS and incubated with acidic medium for 6 h before subsequent experiments.

### Animal model and in vivo angiogenic assay

Femoral artery ligation (FAL) and in vivo angiogenic assays were performed as described in previous studies [17]. In brief, mice were anesthetized with ketamine and xylazine, and their hind limbs were removed. The temperature of the body was maintained at 37.0 ± 0.5 °C. The left femoral artery was exposed through a 2-mm incision without retraction and with minimal tissue disturbance. A 7–0 ligature was placed distal to the origin of the lateral caudal femoral and superficial epigastric arteries and proximal to the genu artery (below the inguinal ligament). The femoral artery was transected between the sutures and separated 1–2 mm, washing the wound with saline and seal. Finally, buprenorphine (0.05–0.1 mg/kg) was administered.

To assess the angiogenic capacity of the cultured EPCs, EPCs (5 × 10^5^ cells) of different groups, including the GPR4 upregulation group and GPR4 baseline group, were resuspended in 100–200 μl of prewarmed PBS (37 °C) and transplanted into the 5 sites near the ligated artery. The same volume of PBS was injected into placebo mice as well. Detection of hindlimb subcutaneous blood flow was performed using a laser Doppler imager (Moor Instruments, Milwey, United Kingdom). Blood flow in both ischemic and nonischemic hindlimbs was measured at 0, 3, 7, 14, and 21 days after the injection. After 21 days, the mice were sacrificed. The limb loss score was calculated by the following rating: 0 = none, 1 = tip necrosis, 2 = toe necrosis, 3 = foot necrosis, 4 = leg necrosis, and 5 = whole leg loss.

### Immunohistochemistry and immunofluorescence analysis

Immunohistochemical staining was performed as described previously. Briefly, right femoral artery ligation and superficial femoral artery (SFA) excision were performed. Rehydration and antigen retrieval were carried out by microwave oven heating in 0.01 M sodium citrate buffer (pH 6.0). Sections were incubated with rabbit monoclonal PECAM-1 antibody (Cell Signaling Technology, MA, USA) followed by HRP Anti-Rabbit DAB Detection kit (Cell Signaling Technology) according to the manufacturers’ instructions. Immunohistological images were acquired using a light microscope (Olympus BX63). Vascular density was determined by counting the number of blood vessels in immunohistochemical staining of CD31 sections and was expressed as vessels per mm^2^.

For immunofluorescence analysis, all sections were incubated with primary antibody (PECAM-1 from Cell Signaling Technology. 1:500) overnight at 4 °C, followed by staining with GFP-conjugated goat anti-rabbit secondary antibody (Abcam, Cambridge, UK). To evaluate cell proliferation in different groups, 5-bromo-2¢-deoxyuridine (BrdU, 50 mg/kg body weight) (Sigma-Aldrich, St. Louis, MO, USA) was injected subcutaneously twice daily for 2 weeks after the surgery. Tissue sections were also stained with Cy5-conjugated anti-BrdU antibodies (Sigma) for capillary density assessment. Nuclei were counterstained with DAPI. Immunofluorescence images were acquired using a confocal microscope (LSM710, Carl Zeiss, GPF: excitation/emission 488/507 nm, Cy5: excitation/emission 633/670 nm).

### Real-time PCR assay

Total RNA was extracted with a High Pure RNA Isolation Kit (Roche, Indianapolis, USA). First-strand cDNA was synthesized using the PrimeScript® RT reagent Kit (Takara Biotechnology, Japan). The mRNA expression of the family was quantified using the 2^−ΔΔCT^ analytical method in triplicate using a StepOne Plus real-time PCR System (ABI, USA). The primers for PCR of GPR4 were as follows: forward 5′-AACCTCTATCGGGTGTTCGTG-3′ and reverse 5′-TTGGCTGTGCTGTTCCTCTTGG-3′. GAPDH was amplified as an internal standard. The relative RNA level in each sample was determined by the 2(−Delta Delta C(T)) method.

### Droplet digital PCR assay

Droplet digital PCR (ddPCR) and data analysis based on EvaGreen Chemistry were performed according to the manufacturer’s instructions. The Supermix (2X) was purchased from Bio-Rad (USA). The final concentration of the primers in the PCR was 200 nM, and the final reaction volume was 20 μL. Each reaction had 1 μL of cDNA, and 1 μL of nuclease-free water was added in place of cDNA for NTC. The BioRad Automated Droplet Generator is used to generate droplets. The following PCR conditions were used: 95 °C for 5 min, 50 °C for 30 s, 56.5 °C for 30 s, and 72 °C for 45 s. The final extension was carried out at 72 °C for 5 min, and then, the signal stabilization step was carried out at 4 °C for 5 min and at 90 °C for 5 min. After PCR, the droplets were analyzed using a QX200 Droplet Reader, and the data were analyzed by using Bio-Rad QuantaSoft software.

### Western blot analysis

Cells were lysed in RIPA buffer (Beyotime, Shanghai, China) supplemented with 1-mM PMSF. Protein extracts were subjected to SDS–PAGE and transferred to polyvinylidene fluoride membranes (Roche, Indianapolis, IN, USA). The following antibodies were used: rabbit anti-GPR4 antibody (1:250; Thermo Fisher Scientific), anti-VEGFA antibody (1:1000; Cell Signaling Technology), and rabbit anti-GAPDH antibody (1:1000; Cell Signaling Technology). Proteins were visualized with HRP-conjugated anti-rabbit IgG (1:3000; Cell Signaling Technology), followed by the use of the ECL chemiluminescence system (Cell Signaling Technology).

### Statistical analysis

All results are expressed as the mean value ± SD. Statistical significance was evaluated by Student’s *t* test or ANOVA. *P* < 0.05 was considered statistically significant. All statistical analyses were performed using SPSS statistical software (SPSS version 21.0).

## Results

### Subject characteristics

**Fig. 1:**
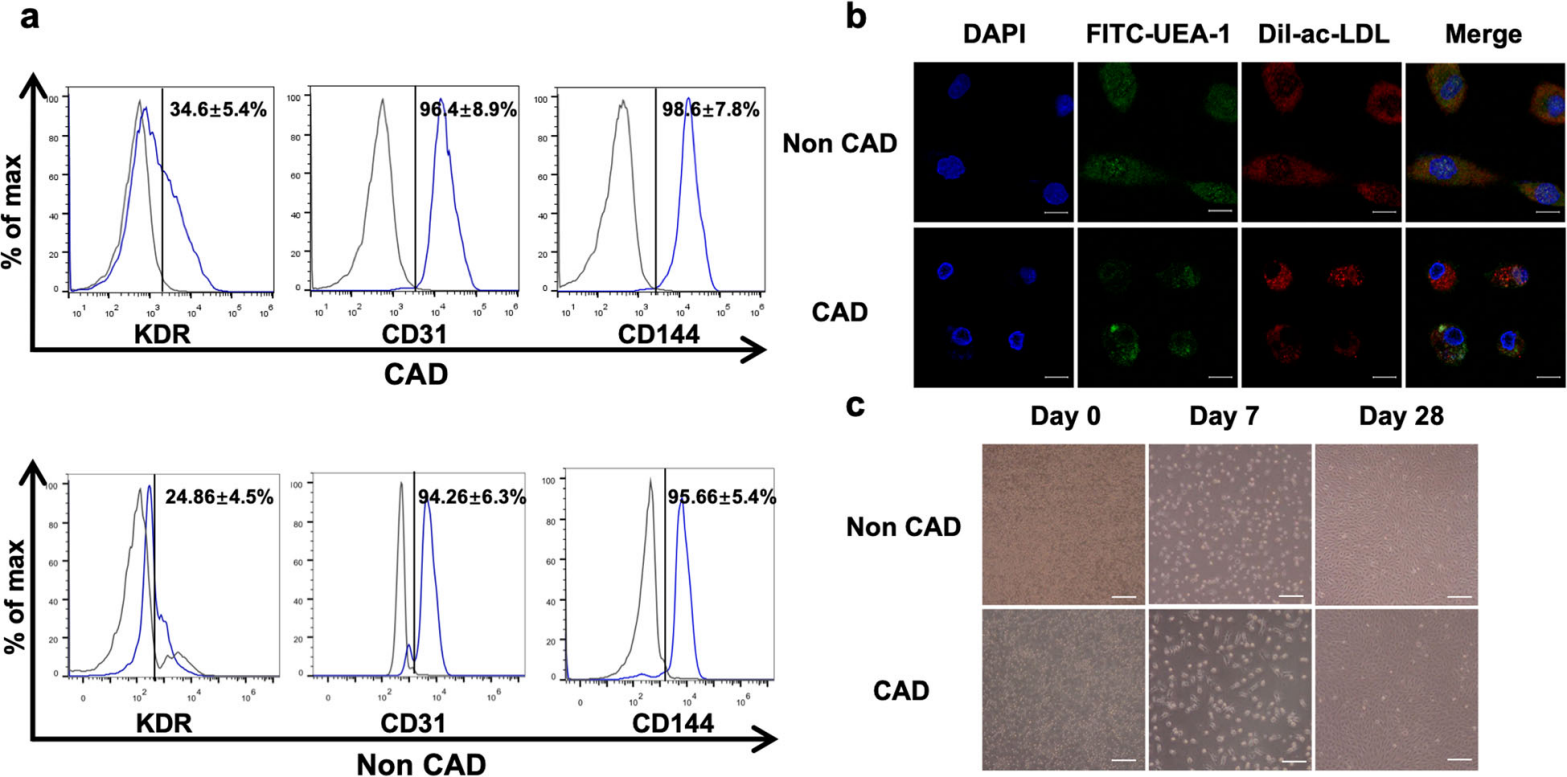
Phenotypic characterization of EPCs after 28 days of culture. **a** Cell surface markers of EPCs as determined by flow cytometry. **b** EPCs were dually positive for DiI-acLDL uptake and FITC-labeled UEA-1 binding. Nuclei were stained with DAPI (blue). Scale bar = 20 μm. **c** Sequential morphological changes of circulating EPCs at different time points after plating (day 0, day 7, and day 28). Scale bar = 100 μm

In total, 20 individuals were enrolled in the study, with 10 individuals in both the CAD and healthy groups. The baseline characteristics are listed in Table S1. There were no significant differences between the two groups at baseline. Late EPCs were obtained by PBMNCs cultured for 28 days (Fig. 1c), similar to previous studies [16]. After 28 days of culture, marker proteins, including KDR, CD31, and CD144, of cultured EPCs were analyzed by flow cytometry analysis (Fig. 1a), and EPCs were dually positive for DiI-acLDL uptake and FITC-labeled lectin-1 binding (Fig. 1b).

### Different EPCs have different angiogenic capacities in an acidic environment

**Fig. 2:**
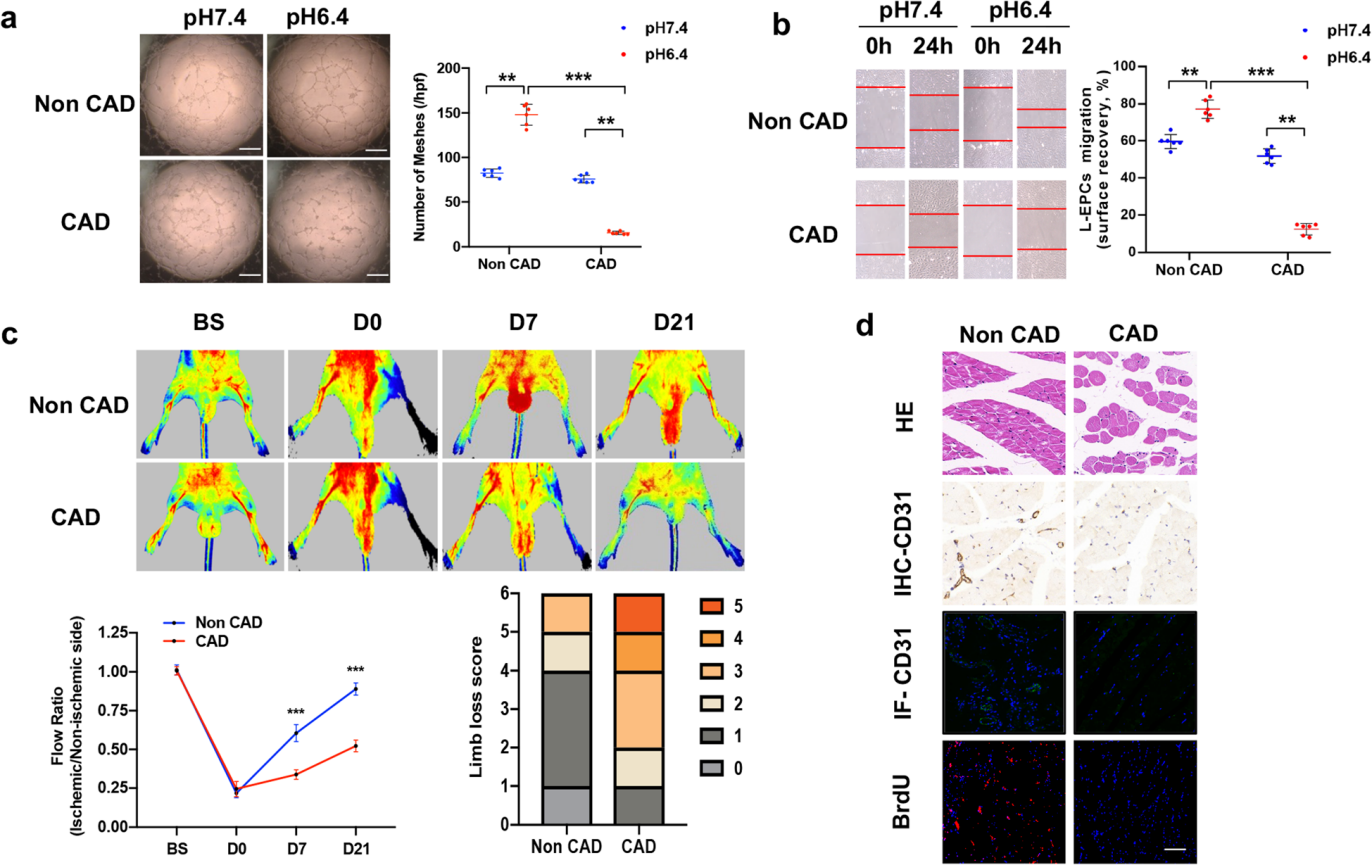
Effect of different pH environments on the angiogenesis of EPCs derived from non-CAD and CAD individuals. **a**, **b** Representative photographs and quantitative analyses of tube formation (**a** scale bar = 40 μm) and migration (**b** scale bar = 20 μm) of EPCs incubated in different pH environments after 6 h (*n* = 6). **c** Representative photographs and quantitative analyses of EPC-mediated neovascularization derived from non-CAD or CAD in hind limb ischemia. *n* = 6 per group. A paired *t* test was performed to determine the statistically significant differences among groups. **d** HE-, IHC-CD31-, IF-CD31-, and BrdU-stained sections of hind limb ischemia and quantitative analyses of CD31-positive vessels, CD31-positive microvessels and BrdU-positive cells. Scale bar = 20 μm. The data are presented as the mean ± SD. **P < 0.01, ***P < 0.001

To determine the in vitro angiogenic capacities of EPCs from different patients, Matrigel experiments were used. Compared to that in the pH 7.4 environment, the angiogenesis of EPCs in the non-CAD group was significantly enhanced by acid stimulation, while the same treatment yielded the opposite result in the CAD group (Fig. 2a). The result of wound healing was similar (Fig. 2b). To further explore the robustness of these results, EPCs from non-CAD and CAD individuals were systemically injected into nude mice subjected to hind limb ischemia, and the results showed that compared to nude mice injected with EPCs from CAD individuals, nude mice that received EPC injections derived from non-CAD individuals exhibited faster flow recovery (Fig. 2c and Fig. S1). The immunohistochemistry of the hind limb is presented in Fig. 2d and Fig. S3a.

### Expression of proton-sensing G-protein-coupled receptors on EPCs

**Fig. 3:**
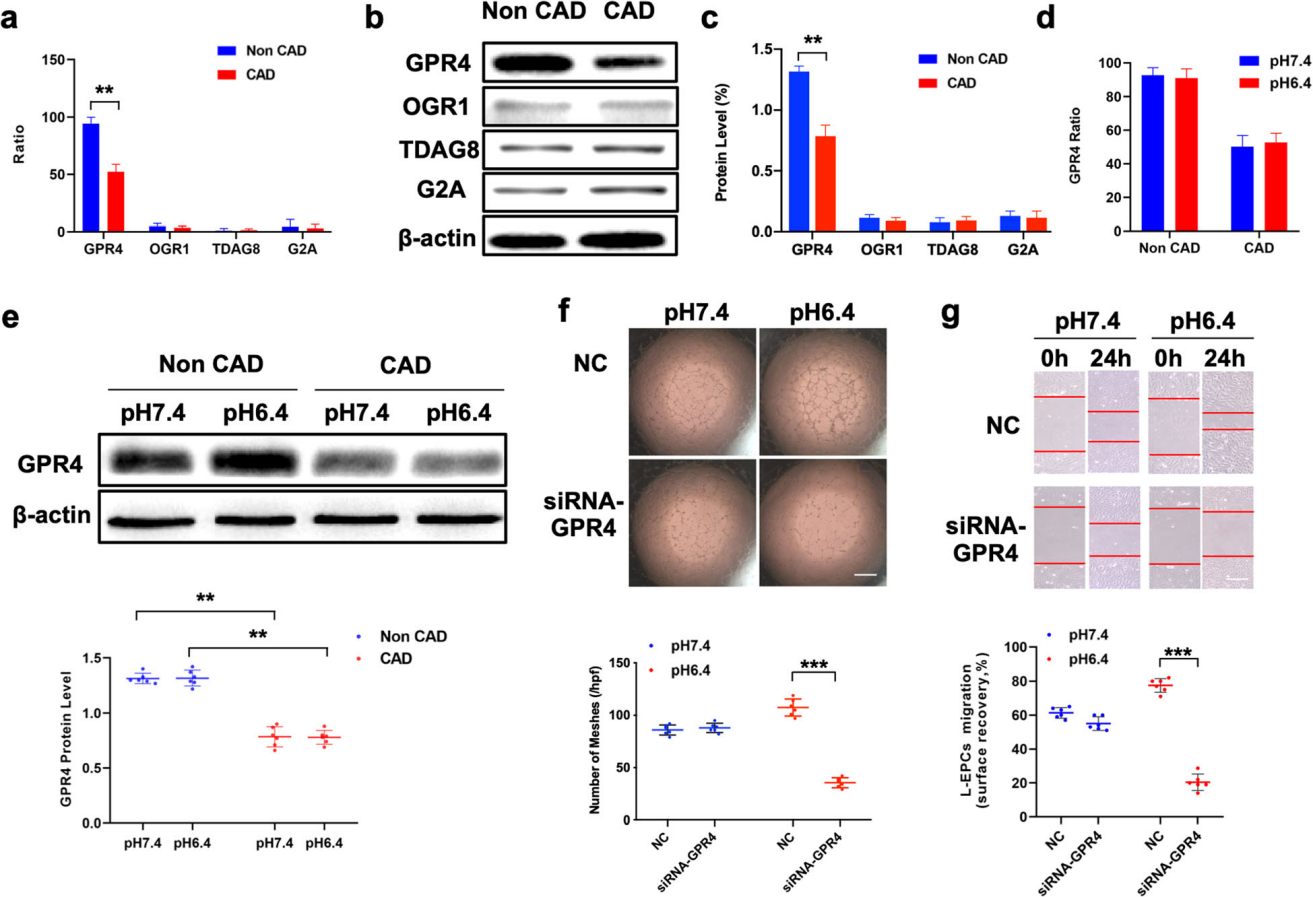
Expression of the proton-sensing GPCR family and angiogenic capacity in EPCs derived from non-CAD and CAD individuals. **a** dPCR was used to determine the mRNA level of proton-sensing GPCR family expression in EPCs (*n* = 6). **b**, **c** Representative photographs and quantitative analyses of the protein levels of the GPCR family (n-6). **d** dPCR was used to determine the mRNA level of GPR4 in different pH environments (*n* = 6). **e** Quantitative analyses and representative photographs of the expression of GPR4 in different pH environments, followed by immunoblotting with a GPR4 antibody. **f**, **g** EPCs were transfected with siRNA-NC or siRNA-GPR4, representative photographs and quantitative analyses of the Matrigel tube-like formation test (scale bar = 40 μm, *n* = 6) and scratch test (scale bar = 20 μm, *n* = 6). The data are presented as the mean ± SD. **P < 0.01, ***P < 0.001

Similar to the study of other cell types using ddPCR, our results showed that all four types of proton-sensing G-protein-coupled receptor subtypes were expressed on EPCs, and GPR4 exhibited the highest expression, which was further confirmed by western blotting (Fig. 3a–c and Fig. S2). Moreover, the ddPCR and western blot results showed that the expression of GRP4 was significantly lower in CAD individuals than in non-CAD individuals in an acidic environment, while the difference between groups did not reach statistical significance regardless of pH (Fig. 3d, e). In EPCs derived from CAD patients, using siRNA-GRP4 to downregulate the expression of GRP4, we found that the in vitro angiogenic capacity of EPCs was impaired in an acidic environment but not in a pH 7.4 environment (Fig. 3f), and the wound healing assay yielded similar results (Fig. 3g).

### GPR4 gene transfer enhanced the angiogenesis of EPCs in an acidic microenvironment

**Fig. 4:**
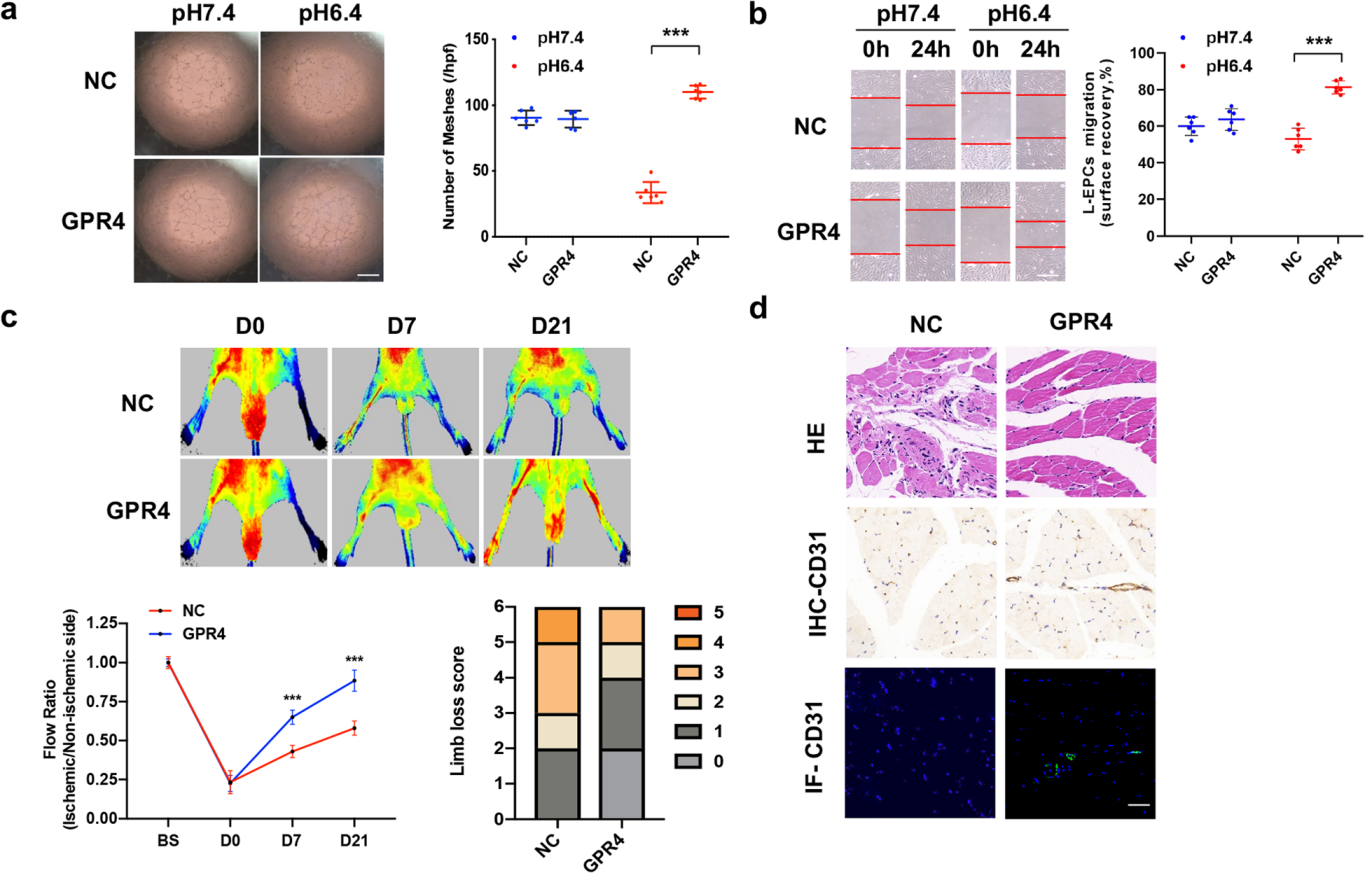
GPR4 gene transfer restored acid-mediated angiogenesis in EPCs derived from CAD patients. **a**, **b** Representative photographs and quantitative analyses of tube formation (**a** scale bar = 40 μm) and migration (**b** scale bar = 20 μm) of EPCs incubated in different pH environments after 6 h (*n* = 6). **c** Representative photographs and quantitative analyses of blood flow in hind limb ischemia in nude mice after injection of EPCs. *n* = 6 per group. A paired *t* test was performed to determine the statistically significant differences among groups. **d** HE, IHC-CD31, and IF-CD31-stained sections of ischemic hind limbs. Scale bar = 20 μm. The data are presented as the mean ± SD. ***P < 0.001

Human GPR4 cDNA was cloned into the eukaryotic expression plasmid. The transcription and expression of GPR4 in EPCs were confirmed by western blot. The pcDNA3.1(+)-GPR4-transduced EPCs exerted a markedly enhanced angiogenesis compared with vector-transduced EPCs in acidic microenvironment, while the difference between groups in the environment of pH 7.4 did not reach statistical significance (Fig. 4a, b).

To determine the effect of GPR4 gene transfer on EPC-mediated angiogenesis in vivo, pcDNA3.1(+)-GPR4-transduced EPCs and vector-transduced EPCs were systemically injected into nude mice subjected to hindlimb ischemia. Remarkably, compared with vector-transduced EPCs, pcDNA3.1(+)-GPR4-transduced EPCs obviously increased hindlimb subcutaneous blood flow in nude mice (Fig. 4c). The immunohistochemistry and immunofluorescence of the hind limb are presented in Fig. S3b and Fig. 4d.

### siRNA-GPR4 decreased the angiogenic capacity of EPCs in an acidic microenvironment

**Fig. 5:**
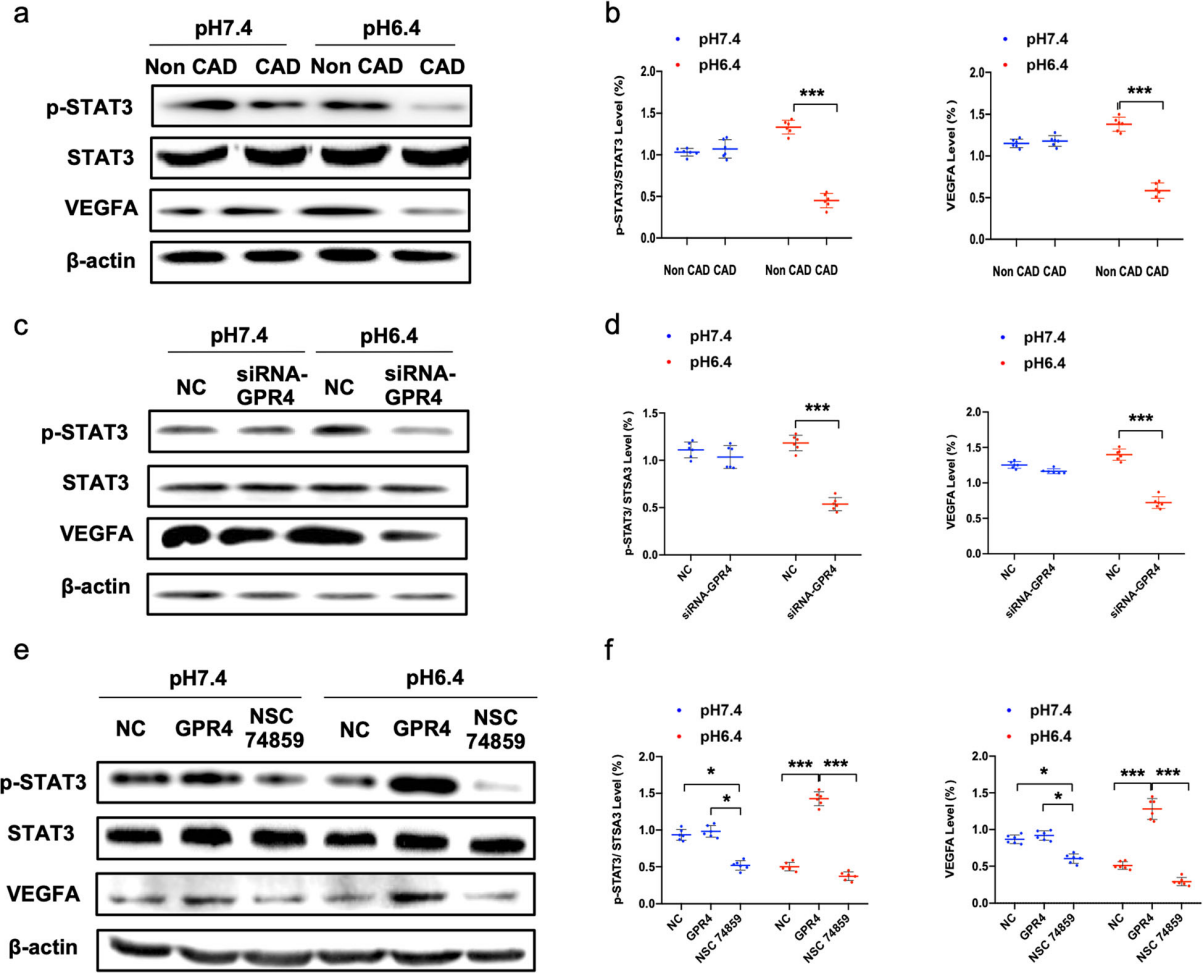
Changes in GPR4 levels influence acid-mediated angiogenesis in EPCs. **a**, **b** Representative photographs and quantitative analyses of p-STAT3, STAT3, and VEGFA protein expression in EPCs derived from non-CAD and CAD individuals in different pH environments (*n* = 6). **c**–**f** Representative photographs and quantitative analyses of p-STAT3, STAT3, and VEGFA protein expression in EPCs derived from non-CAD and CAD individuals in different pH environments. EPCs were transfected with siRNA-GPR4 and negative control siRNA (NC) (*n* = 6) or plasmids expressing GPR4, negative control plasmids (NC), and NSC74589 (*n* = 6). The data are presented as the mean ± SD. *P < 0.05, ***P < 0.001

Furthermore, we investigated the effect of GPR4 signaling pathway activation on the expression of STAT3 phosphorylation (p-STAT3) and VEGFA. Our data showed that at pH 6.4, compared to that in non-CAD individuals, the expression of p-STAT3 and VEGFA was significantly lower in EPCs derived from CAD individuals, while the difference between groups was not significant at pH 7.4 (Fig. 5a, b). To further confirm the angiogenic capability of GPR4 in an acidic microenvironment, we silenced the expression of GPR4 in EPCs with siRNA. The expression of GPR4, STAT3, and VEGFA in EPCs was confirmed by western blot. Our data show that siRNA-GPR4 results in decreased expression of p-STAT3 and VEGFA at pH 6.4 but not at pH 7.4 (Fig. 5c, d).

### Increased in vitro and in vivo angiogenesis after upregulation of GPR4 can be blocked by STAT3 inhibitor

**Fig. 6:**
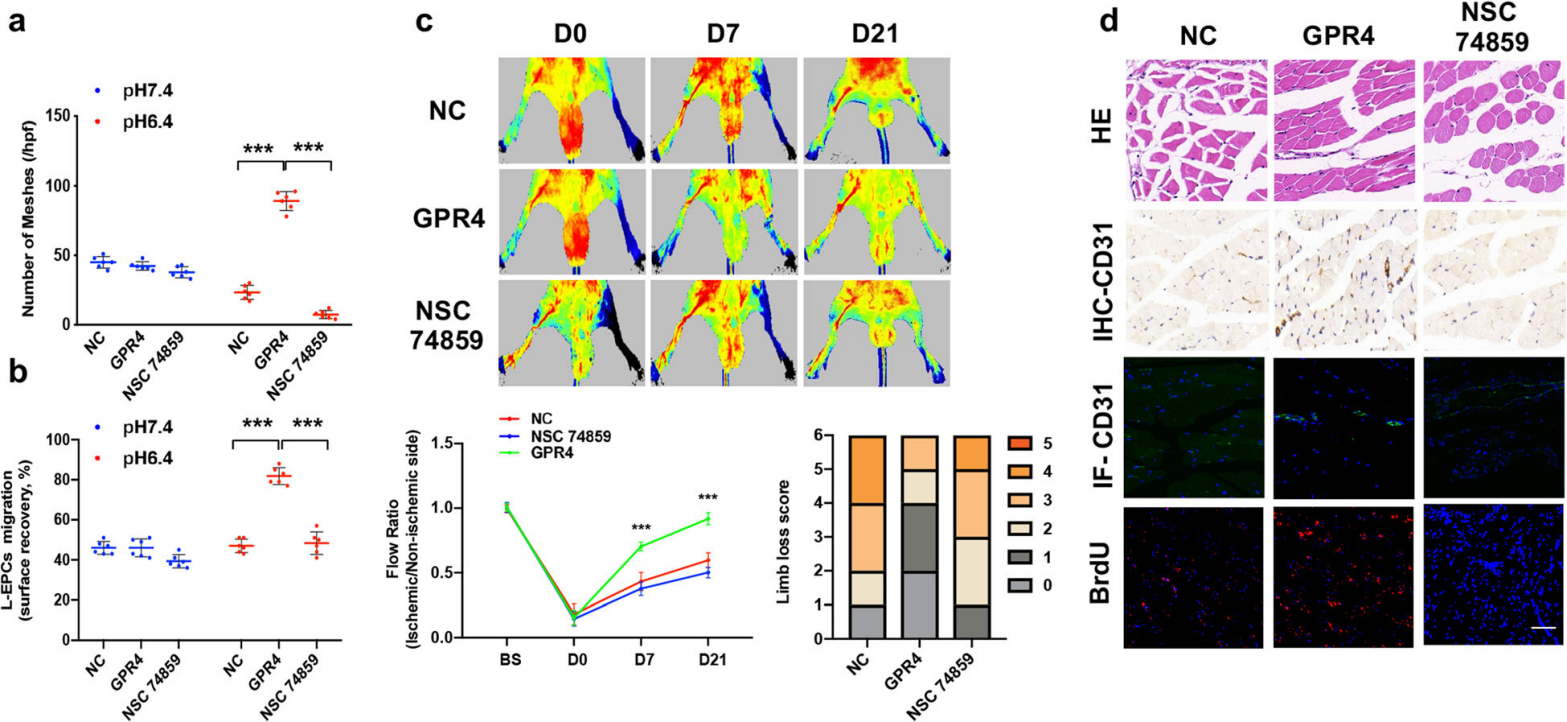
GPR4 regulates VEGFA expression via the STAT3 signaling pathway stimulated by an acidic microenvironment in EPCs derived from CAD individuals. **a**, **b** Quantitative analyses of tube formation (**a**) and migration (**b**) incubated in different pH environments in EGM medium after 6 h (*n* = 6). **c** Representative photographs and quantitative analyses of blood flow in hind limb ischemia in nude mice after injection of EPCs transfected with negative control plasmids (NC) or plasmids expressing GPR4 and NSC74859. *n* = 6 per group. A paired *t* test was performed to determine the statistically significant differences among groups. **d** HE-, IHC-CD31-, IF-CD31-, and BrdU-stained sections of ischemic hind limbs. Scale bar = 20 μm. The data are presented as the mean ± SD. ***P < 0.001

Our data indicated that NSC 74859, an inhibitor of STAT3, can lower VEGFA levels, which were upregulated by GPR4 gene transfer (Fig. 5e, f), and this finding is also parallel to the migration and Matrigel experimental results (Fig. 6a, b). In addition, NSC 74859 eliminated the increased hindlimb subcutaneous blood flow in nude mice caused by GPR4 gene transfection (Fig. 6c). The immunohistochemistry and immunofluorescence of the hind limb are presented in Fig. S3c and Fig. 6d.

## Discussion

Our current study found that the expression of GPR4 in CAD patients was significantly reduced compared with that in healthy subjects, which led to the impaired angiogenic capacity of EPCs in an acidic ischemic environment. The upregulation of GPR4 expression significantly augmented the adaptive angiogenic capacity of EPCs in CAD patients in acidic microenvironments both in vivo and in vitro. Mechanistically, augmentation of GPR4 expression can enhance the binding of H^+^, resulting in increased expression of the downstream STAT3/VEGFA signaling pathway and finally accelerating the angiogenesis of EPCs. GPR4 may be a promising target for EPCs to adjust to acidosis environments and restore impaired angiogenic capacity.

It is generally accepted that EPCs have been an important part of transplantational treatment in vascular repair and regenerative medicine [18, 19]. Although EPC-based experimental trials in CAD are diversely being initiated, controversy exists despite functional improvement being observed in some studies [20, 21]. Among them, EPCs from CAD patients present low amounts and impaired function [22]. Therefore, it is essential to explore the therapeutic pattern of EPCs under acidic conditions and its underlying mechanism.

The acidic microenvironment, as a pathophysiological chemical factor of ischemia, could significantly affect the function of mobilized EPCs [3, 10]. Interestingly, previous data indicated that exposure of EPCs to acidic medium has a dual effect on cell function. It has been widely accepted that an acidic microenvironment exerts an additional destructive effect on stem/progenitor cells because a decrease in extracellular pH induces cellular acidification and subsequently cell death [23]. On the other hand, increasing data revealed that an acidic environment could also provide stimuli that enhanced cellular capacities, which indicated that a severe microenvironment could inspire cell self-adaption/defense to maintain their abilities [24]. Notably, evidence has shown that the vascularization of EPCs from healthy people could be promoted by an acidic microenvironment at the appropriate time [9].

Mounting data have shown that H^+^ can trigger microenvironment-induced effects on various cells, such as cancer cells and endothelial cells, and proton-sensing GPCRs have been suggested as associated receptors to mediate downstream intracellular signaling pathways [25–27]. Wyder et al. [14] showed that tumor growth, cell proliferation and altered vessel morphology, length and density are critically reduced in mice lacking GPR4 compared to wild-type mice. Given the relationship between proton-sensing GPCRs and acidosis, proton-sensing GPCR family members might function in EPCs and deteriorate in EPCs from CAD patients. Interestingly, Ding et al. found that proton sensory protein OGR1 mediated inhibitory effects of proliferation and angiogenesis of EPCs by acid activation [28]. In the present study, we assessed proton-sensing GPCR levels in EPCs, and GPR4 expression was comparatively abundant to OGR1, TDAG8, and G2A, which indicated that EPCs might sense the local acidic microenvironment via GPR4 to adjust themselves to a destructive milieu. Furthermore, the expression of GPR4 was significantly reduced in EPCs from CAD patients compared with those from healthy subjects, consistent with impaired acid-induced neovascularization. Thus, the parallel tendency in both GPR4 expression and angiogenic capacity of EPCs suggests that GPR4 might mediate the abnormality in cell function of EPCs in acidic microenvironment. Our findings provide further evidence that there might be various proton-sensing GPCRs involved in EPCs that might interact with each other in an acidic environment.

It has been reported that GPR4 is overexpressed in tumors and ECs, which can induce an increase in angiogenesis [29, 30]. Interestingly, the overexpression of GPR4 also presented negative cell activities in some research, which indicated that the effect of GPR4 on different types of cells is controversial [31]. Kim et al. reported that SPC/GPR4 interactions induce angiogenesis of ECs in vivo and that the activation of phosphatidylinositol-3 kinase and Akt are both involved, identifying that GPR4 might function as a critical regulator of the angiogenic capacity of ECs [13]. Ren et al. further disclosed that overexpression of GPR4 mediates the cellular extension of Notch1 signaling, and this increment was correlated with increased endothelial tube formation of HMEC-1 cells in vitro [25]. Thus, GPR4 might serve as a target for vascular homeostasis in endothelial cell lines. To further demonstrate how GPR4 functions in EPC-mediated angiogenic capacity in CAD patients, we upgraded the expression of GPR4 by pcDNA3.1(+)-GPR4 and stimulated EPCs to respond to acidic stimuli. Indeed, our results show that pcDNA3.1(+)-GPR4 gene transfer could enhance angiogenesis in EPCs from CAD patients at pH 6.4 compared to the normal microenvironment at pH 7.4. Consistent with this, our data show that the increase in GPR4 expression is parallel to the increased capability of EPC adhesion, migration, and tube formation both in vitro and in vivo at pH 6.4. In addition, siRNA-GPR4 gene transfer induced a decrease in GPR4 expression in EPCs from healthy subjects, and the in vitro and in vivo abilities of EPCs were also downregulated at pH 6.4. However, the functional changes of the EPCs described above were not found at pH 7.4. Therefore, our current study demonstrated that reduced expression of GPR4 was, at least partially, responsible for EPC-mediated angiogenesis decline in CAD under acidic circumstances, providing novel insight into the molecular mechanism for our understanding of EPC angiogenic dysfunction in acidic microenvironments.

The signal transducer and activator of transcription (STAT) family serves as cytoplasmic transcription factors that promote the intracellular signal pathway response to cytokines and factors [32]. It is composed of seven members: STAT1, STAT2, STAT3, STAT4, STAT5A, STAT5B, and STAT6. STAT3 has been suggested to enhance cell growth and survival, angiogenesis, migration, invasion, or metastasis and has been reported to be regulated by GPCRs [33–35]. Previous research showed that angiogenesis and tumor growth of breast cancer cells could be attenuated by inhibiting STAT3-VEGF expression both in vitro and in vivo in an acidic tumor environment [36, 37]. Moreover, STAT3-induced angiogenesis was also demonstrated in other types of cells, such as hepatocellular carcinoma [38]. Thus, we proposed the hypothesis that STAT3 could be one of the vital steps of the signaling pathway in acid-related angiogenesis of EPCs. However, whether STAT3 activates acid-mediated neovascularization remains unknown. In our study, we found that transfection of GPR4 improved angiogenic capacity and augmented the expression of VEGFA in EPCs from CAD patients, which was inhibited by knockdown of GPR4. Additionally, our data showed that the ratio of p-STAT3/STAT3 was increased, consistent with the increase in VEGFA expression and the alteration of GPR4 expression. These results suggest that there might be a correlation between GPR4-mediated acid-induced angiogenesis and the STAT3/VEGFA pathway. Furthermore, the increased EPCs in vitro and in vivo function in acidic microenvironment-mediated angiogenesis was inhibited when STAT3 was blocked. These results revealed that the GPR4/STAT3/VEGFA signaling pathway was involved in EPC functional regulation in the acidic microenvironment.

However, there are some limitations in our present study. First, the acidic microenvironment is a complex microsystem composed of multiple proton-sensing proteins. The linkage of GPR4 signaling with other receptors needs further investigation. Moreover, the GPR4 signaling pathway related to the incidence and prognosis of CAD also deserves to be elucidated.

## Conclusions

In conclusion, our present study demonstrated for the first time that the GPR4/STAT3/VEGFA signaling pathway contributed to the impaired angiogenic capacity of EPCs and that elevating GPR4 could restore the angiogenic capacity of EPCs from CAD patients. These findings provide a novel cell-based therapeutic target for CAD.

## Supplementary Information

**Additional file 1: Supplemental Fig. S1**. Representative photograph and quantitative analyses of the pH value in the ischemic hind limb of nude mice.

**Additional file 2: Supplemental Fig. S2**. a, RT-PCR was used to determine the mRNA levels of proton-sensing GPCR family members in EPCs (*n* = 6). b, RT-PCR was used to determine the mRNA level of GPR4 in different pH environments (n = 6).

**Additional file 3: Supplemental Fig. S3**. a, Quantitative analyses of IHC-CD31-, IF-CD31- and BrdU-stained sections of ischemic hind limbs in Fig. 2. b, Quantitative analyses of IHC-CD31- and IF-CD31-stained sections of ischemic hind limbs in Fig. 4. c, Quantitative analyses of IHC-CD31-, IF-CD31- and BrdU-stained stained sections of ischemic hind limbs in Fig. 6. The data are presented as the mean ± SD. ***P < 0.001.

## Data Availability

All data generated or analyzed during this study are included in this manuscript.

## Abbreviations

ANOVA: Analysis of variance
ASIC1a: Acid-sensing ion channel 1a
CAD: Coronary artery disease
CD31: Platelet endothelial cell adhesion molecule-1
cDNA: Complementary DNA
ddPCR: Droplet digital PCR
EBM-2: Endothelial cell basal medium-2
EPCs: Endothelial progenitor cells
FAL: Femoral artery ligation
GAPDH: Glyceraldehyde-3-phosphate dehydrogenase
GPR4: G-protein-coupled receptor 4
GPCRs: Proton-sensing G-protein-coupled receptors
HEPES: *N*-2-hydroxyethylpiperazine-*N*′-2-ethanesulfonic acid
KDR: Kinase-insert domain receptor
PBMNCs: Peripheral blood mononuclear cells
PBS: Phosphate-buffered saline
p-STAT3: Phospho-signal transducer and activator of transcription 3
PKA: Protein kinase A
RIPA: Radioimmunoprecipitation assay
SD: Standard deviation
siRNA: Short interfering RNA
STAT3: Signal transducer and activator of transcription 3
VEGFA: Vascular endothelial growth factor A
