# Supplementary figures and images for: GPR4 signaling is essential for the promotion of acid-mediated angiogenic capacity of endothelial progenitor cells by activating STAT3/VEGFA pathway in patients with coronary artery disease
Source: Stem Cell Res Ther. 2021 Feb 25;12:149. doi: 10.1186/s13287-021-02221-z (PMC7905863; doi:10.1186/s13287-021-02221-z)

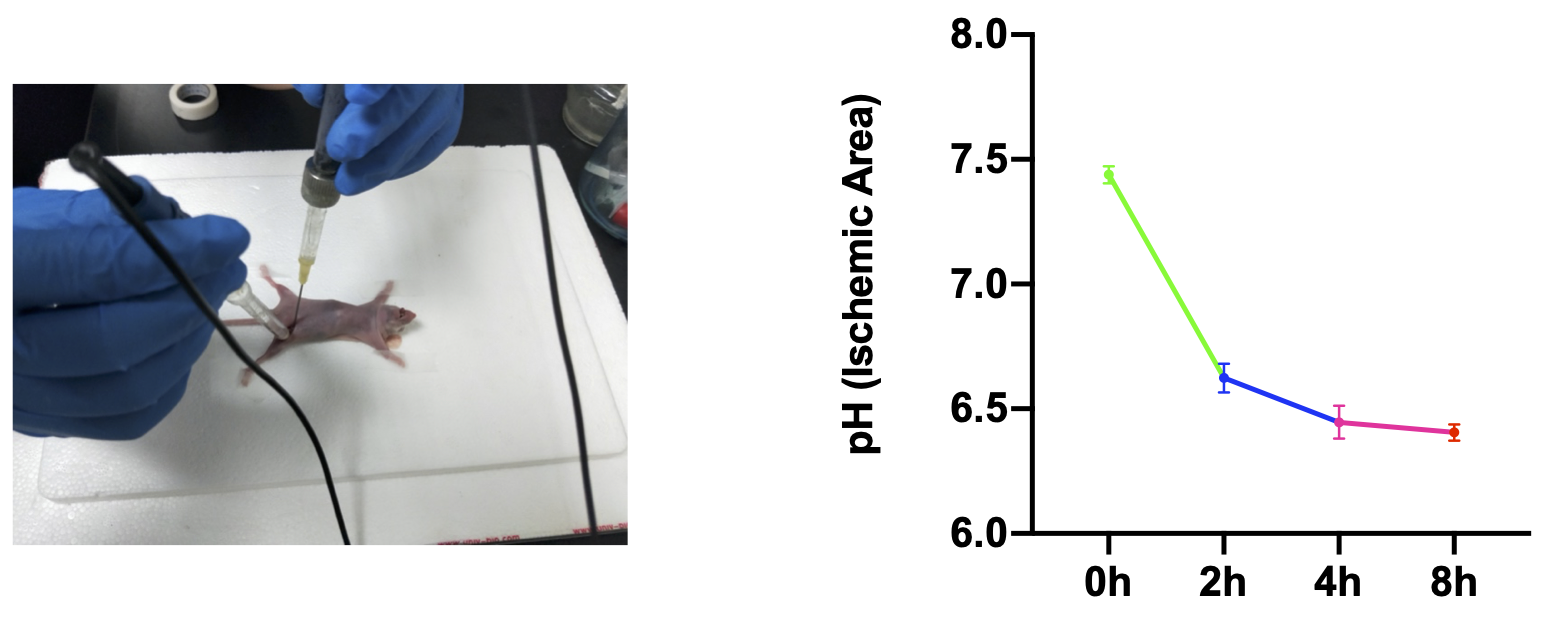

Supplement: Supplementary file 1 — Additional file 1: Supplemental Fig. S1. Representative photograph and quantitative analyses of the pH value in the ischemic hind limb of nude mice. [file 13287_2021_2221_MOESM1_ESM.tiff]

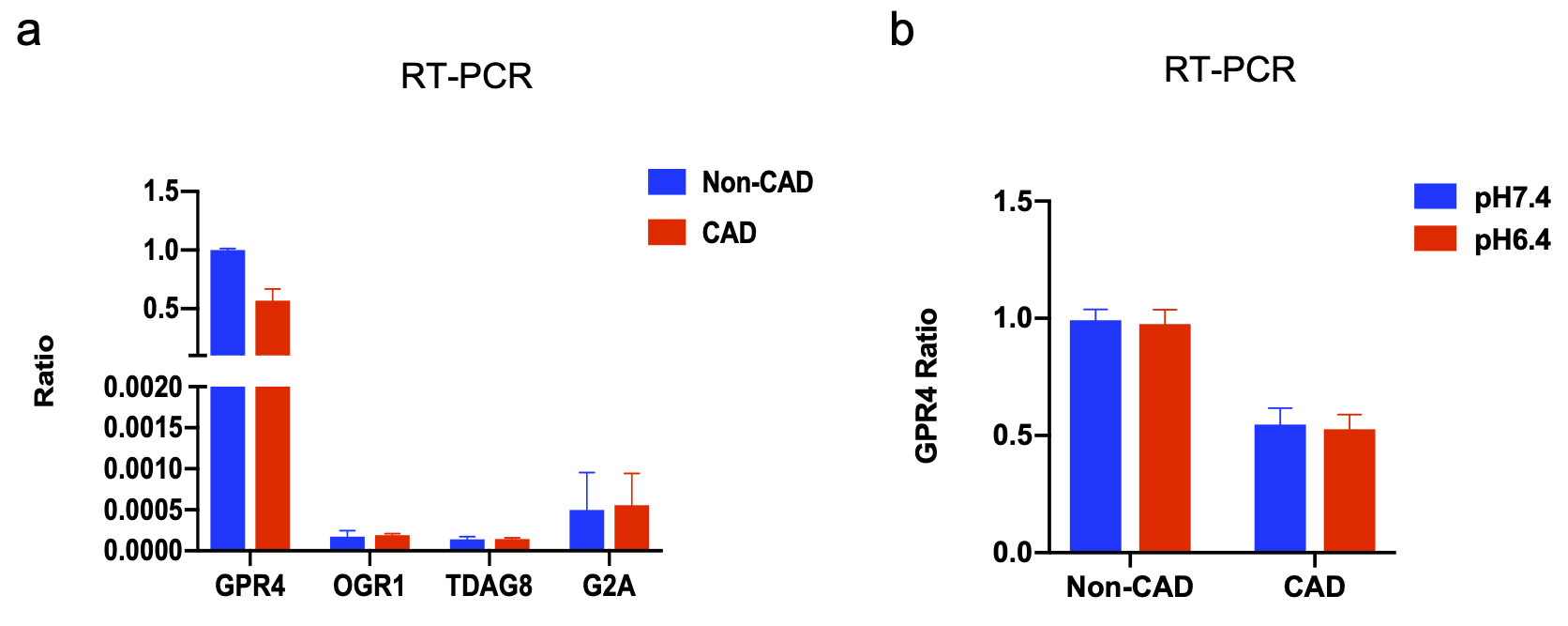

Supplement: Supplementary file 2 — Additional file 2: Supplemental Fig. S2. a, RT-PCR was used to determine the mRNA levels of proton-sensing GPCR family members in EPCs (n = 6). b, RT-PCR was used to determine the mRNA level of GPR4 in different pH environments (n = 6). [file 13287_2021_2221_MOESM2_ESM.tiff]

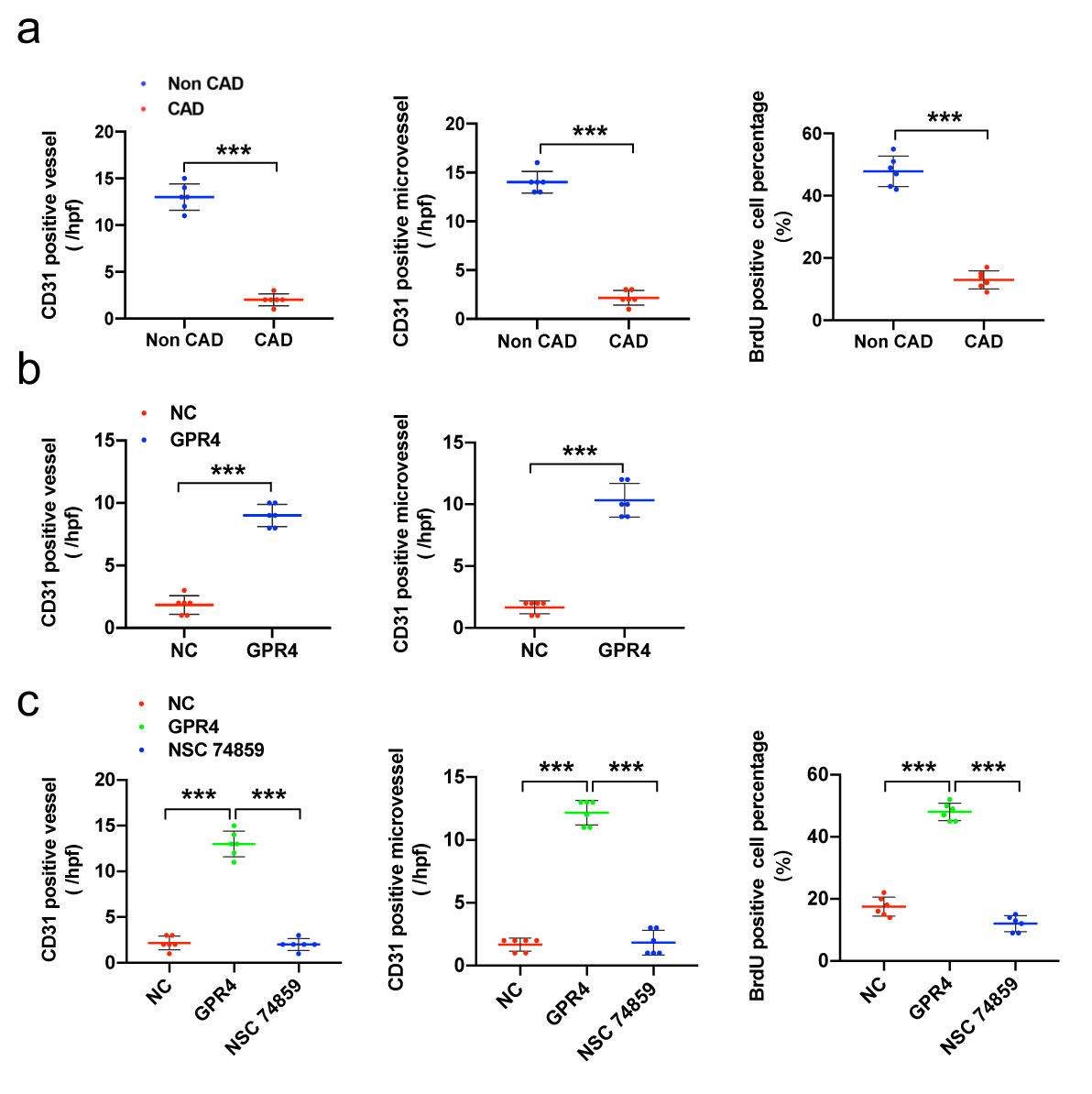

Supplement: Supplementary file 3 — Additional file 3: Supplemental Fig. S3. a, Quantitative analyses of IHC-CD31-, IF-CD31- and BrdU-stained sections of ischemic hind limbs in Fig. 2. b, Quantitative analyses of IHC-CD31- and IF-CD31-stained sections of ischemic hind limbs in Fig. 4. c, Quantitative analyses of IHC-CD31-, IF-CD31- and BrdU-stained stained sections of ischemic hind limbs in Fig. 6. The data are presented as the mean ± SD. ***P < 0.001. [file 13287_2021_2221_MOESM3_ESM.tiff]
